# Major Depressive Disorder With Non‐Suicidal Self‐Injury in Adolescents: Altered Morphological Inverse Divergence Gradient in Brain Structure and Gene Expression Patterns

**DOI:** 10.1002/hbm.70653

**Published:** 2026-10-21

**Authors:** Yan Zhang, Xinyuan Hu, Wei Wang, Jing Wang, Hongying Li, Yumei Wan, Jianlei Zhu, Fei Liu, Shiyue Tao, Duanwei Wang, Xiangtao Lin, Yingying Zhang, Hongmei Zhang, Yuandong Gong

**Affiliations:** ^1^ Shandong Mental Health Center Shandong University Jinan Shandong China; ^2^ Shandong Provincial Key Medical and Health Laboratory of Digital Psychiatry, Shandong Mental Health Center Shandong University Jinan Shandong China; ^3^ The Second Veterans Hospital of Sichuan Chengdu Sichuan China; ^4^ School of Information Science and Engineering University of Jinan Jinan China; ^5^ Cheeloo College of Medicine Shandong University Jinan Shandong China; ^6^ Medical Management Service Center of Health Commission of Shandong Province Jinan Shandong China

**Keywords:** adolescent, gene expression, major depressive disorder, morphometric inverse divergence gradient network, neurotransmitter receptors, non‐suicidal self‐injury

## Abstract

Adolescents with major depressive disorder (MDD) and non‐suicidal self‐injury (NSSI) face serious challenges to their mental and physical health. The development of this condition is influenced by genetic, environmental, and brain development factors. In this study, we used the Morphometric Inverse Divergence Gradient Network (MIND‐Gd) for the first time to explore brain structural network abnormalities and their transcriptional mechanisms in adolescents with MDD and NSSI. A total of 241 adolescents with MDD participated in this study. Among them, 134 had NSSI, while 107 did not. Using high‐resolution T1‐weighted magnetic resonance imaging (MRI), we constructed the principal MIND gradient network. This network reflects the similarity of cortical morphology between different brain regions. The results showed that adolescents with NSSI had higher MIND gradient values in the left caudal middle frontal gyrus and bilateral inferior temporal gyri. In contrast, lower values were observed in the left lingual gyrus and right superior parietal lobule. These structural changes were spatially correlated with functional alterations in the dorsal attention network. Further analysis revealed that these differences were linked to the distribution of nine types of neurotransmitter receptors, including 5‐HT1A, GABAa, glutamate, and dopamine. Partial least squares regression analysis (PLS) was also conducted. The first component (PLS1) explained 39.53% of the spatial variance in MIND‐Gd. This analysis identified key genes related to NSSI, such as GABRA5 and MGST1. Enrichment analysis showed that PLS1+ genes were involved in synaptic signaling, neurodevelopment, and mood disorder pathways. These genes were enriched in astrocytes and cortical layers I, II, and V. On the other hand, PLS1− genes were linked to chromatin regulation and cytoskeletal metabolism. These genes were mainly expressed in Cortical Layers III and IV. In conclusion, adolescents with MDD and NSSI show specific abnormalities in brain structural gradients. These abnormalities are closely related to neurotransmitter receptor distributions and gene expression patterns. The findings provide multi‐level evidence for understanding the neurobiological mechanisms of NSSI.

## Highlights

1. First use of MIND‐Gd network in adolescents with MDD and NSSI reveals altered structural gradients.
2. Gradient changes spatially correlate with neurotransmitter receptor distributions and key synaptic/neurodevelopmental genes.
3. Distinct gene expression patterns are seen across cortical layers and astrocytes.

## Graphical abstract

Using the morphometric inverse divergence gradient network (MIND‐Gd), this study shows that adolescents with major depressive disorder (MDD) and non‐suicidal self‐injury (NSSI) exhibit altered principal structural gradients relative to adolescents with MDD without NSSI, and that these gradient changes are spatially coupled to neurotransmitter receptor distributions and to the expression of synaptic and neurodevelopmental genes across cortical layers and cell types.

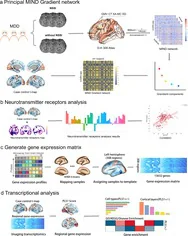

**Abbreviations**
AHBA: Allen Human Brain Atlas
Cluster‐FDR: cluster‐level false discovery rate
CT: cortical thickness
DAN: dorsal attention network
DWI: diffusion weighted imaging
FDR: false discovery rate
GLM: general linear model
GMV: gray matter volume
ICD‐10: International Classification of Diseases, 10th Revision
MC: mean curvature
MDD: major depressive disorder
MIND: morphometric inverse divergence
MIND‐Gd: morphometric inverse divergence gradient
MRI: magnetic resonance imaging
MSN: morphometric similarity network
NSSI: non‐suicidal self‐injury
PLS: partial least squares regression
PLS1+/PLS1− Genes: PLS component 1 positive/negative weight genes
SA: surface area
SCN: structural covariance networks
SD: sulcal depth

## 1 Introduction

Major depressive disorder (MDD) is a chronic, recurrent psychological disorder caused by multiple factors, including genetic, environmental, and neurobiological influences. It is not only a leading cause of global disability but also a core contributor to the disease burden among adolescents (Filatova et al. 2021; Gore et al. 2011). The prevalence of non‐suicidal self‐injury (NSSI) is significantly higher in adolescents with MDD, with studies showing that approximately 40%–60% of these patients engage in NSSI (Li et al. 2024; Hu et al. 2024). Although NSSI is defined as deliberate self‐inflicted damage to body tissues without suicidal intent, more than 66% of adolescents who attempt suicide have a history of NSSI (Hooley et al. 2020; Voss et al. 2020). Epidemiological studies further reveal that NSSI characteristics and prevalence among adolescents are consistent across different countries (Plener et al. 2009; Brown and Plener 2017). This global phenomenon poses a serious threat to adolescents' physical and mental health and is considered one of the strongest predictors of suicidal behavior (Mars et al. 2019; Mannekote Thippaiah et al. 2021; Lawrence et al. 2023).

In adolescent MDD patients, NSSI can often be identified through self‐harming behaviors and visible scars. While these outward manifestations provide clues for clinical diagnosis, they fail to capture the underlying neurobiological mechanisms driving such behaviors. Therefore, studying brain structure abnormalities is important for understanding the disease mechanisms and informing more effective therapeutic strategies. Many studies have shown that MDD and NSSI are linked to abnormalities in cortical structure and disrupted brain networks (Pang et al. 2024; Zhang et al. 2025; Wu et al. 2024), particularly evident in the structural connectome (Xue et al. 2023, 2025). The structural connectome, representing the brain's large‐scale network of anatomical connections, plays a vital role in coordinating cognitive, emotional, and behavioral functions. Despite these advances, research on specific structural connectome abnormalities in MDD patients with NSSI remains limited. The differences between these patients and MDD patients without NSSI also remain unclear. Addressing these gaps is essential for advancing our understanding of the distinct neural mechanisms underlying NSSI in adolescent MDD. Structural brain abnormalities in adolescent MDD and NSSI have been consistently demonstrated using various morphometric approaches (Yang et al. 2025).

Several methods have been applied to analyze brain structural networks in MDD and NSSI in recent years (Zielinski et al. 2010; Singh et al. 2013; Repple et al. 2020). These include white matter network analysis based on diffusion weighted imaging (DWI) (Hu et al. 2024), structural covariance networks (SCN) (Zielinski et al. 2010; Singh et al. 2013; Repple et al. 2020), morphometric similarity networks (MSN) (Li et al. 2021), and morphometric inverse discrete networks (MIND) (Sebenius et al. 2023). While each method has contributed significantly, they also present notable limitations. DWI fiber tractography, although capable of objective quantitative assessment, has drawbacks when comparing the strength of long‐distance links (Donahue et al. 2016). SCN works well in group‐level analysis but fails in individualized characterization (Alexander‐Bloch et al. 2013; Wang and He 2024). Dynamic time warping mapping is applicable to individualized analysis but relies heavily on longitudinal cohort data (Sun et al. 2024). MSN maps multimodal structural features into structural vectors and connects morphometric measures with connectomic networks, providing theoretical support for research on brain structure and cortical gene expression. However, MSN simplifies rich cortical vertex data into a single statistical measure, inevitably losing information, and assumes uniform cortical morphometric variability, which reduces network stability. In contrast, the recently proposed MIND approach measures the divergence of five morphometric measurements at the vertex level to construct morphometric similarity networks. Compared to earlier methods, MIND is more stable, offers better cortical matching, and shows stronger genetic relationships. A key advantage is that it requires only T1‐weighted MRI data to construct reliable networks, making MIND more practical. Furthermore, early brain partitioning and cortical parcellation were guided by morphogenetic gradients, which quantify the spatial organization of cortical structures along continuous axes. This captures key features of cortical organization, including gene expression, cell structure, myelin distribution, and evolutionary growth, thus providing more detailed structural network information (Huntenburg et al. 2018; Yang et al. 2021).

MDD is a clinically heterogeneous disorder with numerous genetic variants, some of which are important in presynaptic regulation and cognitive function (Havinga et al. 2017; Meng et al. 2024; Liu, Wang, et al. 2024). Researchers have used whole‐brain gene expression data from the Allen Human Brain Atlas to link macro‐structural brain disorders with specific transcriptional expression patterns (Li et al. 2021; Anderson et al. 2020; Wang et al. 2024). Recent studies have identified significant differences in gene expression, enriched pathways, and expression patterns in the MSN network space of MDD patients (Xue et al. 2025), suggesting that gradient similarity network research is necessary to recognize neuroimaging biomarkers and assess genetic impacts. However, most of these studies investigated MSN networks. For NSSI patients, abnormal mechanisms and their dependence on disease‐related gene expression remain underexplored. The MIND network exhibits higher biological validity and can more accurately reflect the coupling between cortical structure and gene co‐expression.

Crucially, prior large‐scale studies have confirmed that MDD leads to replicable brain structural network changes relative to healthy controls. For example, Li et al. found that cortical differences in MDD correlate with cell‐type‐specific gene expression (Li et al. 2021). Qin et al. showed that MDD patients with suicidal thoughts exhibit network deficits linked to synaptic signaling (Qin et al. 2024). Kang et al. identified gray matter and functional abnormalities in MDD patients with NSSI compared to healthy controls (Kang et al. 2022). These findings confirm that MDD patients deviate from normative brain development at the connectome level. Based on this evidence, the present study focuses on comparing MDD patients with NSSI versus those without NSSI to explore whether additional connectomic disruptions are specifically associated with NSSI beyond common MDD‐related alterations.

This study aims to construct a new MIND gradient network to evaluate differences in brain structural network gradient changes between NSSI group and non‐NSSI group. Using transcriptomic data, we explore how molecular organization and microstructural changes in brain morphometric coordination affect brain morphometry in MDD patients with NSSI. Specifically, we measure MIND cortical morphometric similarity at regional and global levels to formulate principal MIND gradients (MIND‐Gd). We examine differences in the hierarchical structure of brain structural network gradients between NSSI and non‐NSSI patients. These gradients are further mapped onto brain functional networks and cortical cellular networks to investigate potential functional implications and cellular‐level changes. Additionally, we analyze abnormal neurochemical signaling associated with principal MIND gradient changes. To further elucidate molecular mechanisms, we compared principal MIND gradient changes to in vivo receptor distribution maps generated with the JuSpace toolbox. We then performed PLS regression analysis to link principal MIND gradient changes to anatomical gene expression patterns and identified closely related genes. Permutation tests detected strongly associated genes. Finally, we conducted gene ontology pathway analysis, cell‐type enrichment analysis, and cortical layer enrichment analysis to validate whether transcriptional changes are consistent with principal MIND gradient alterations. Figure 1 presents the overall analytical framework of this study.

**FIGURE 1:**
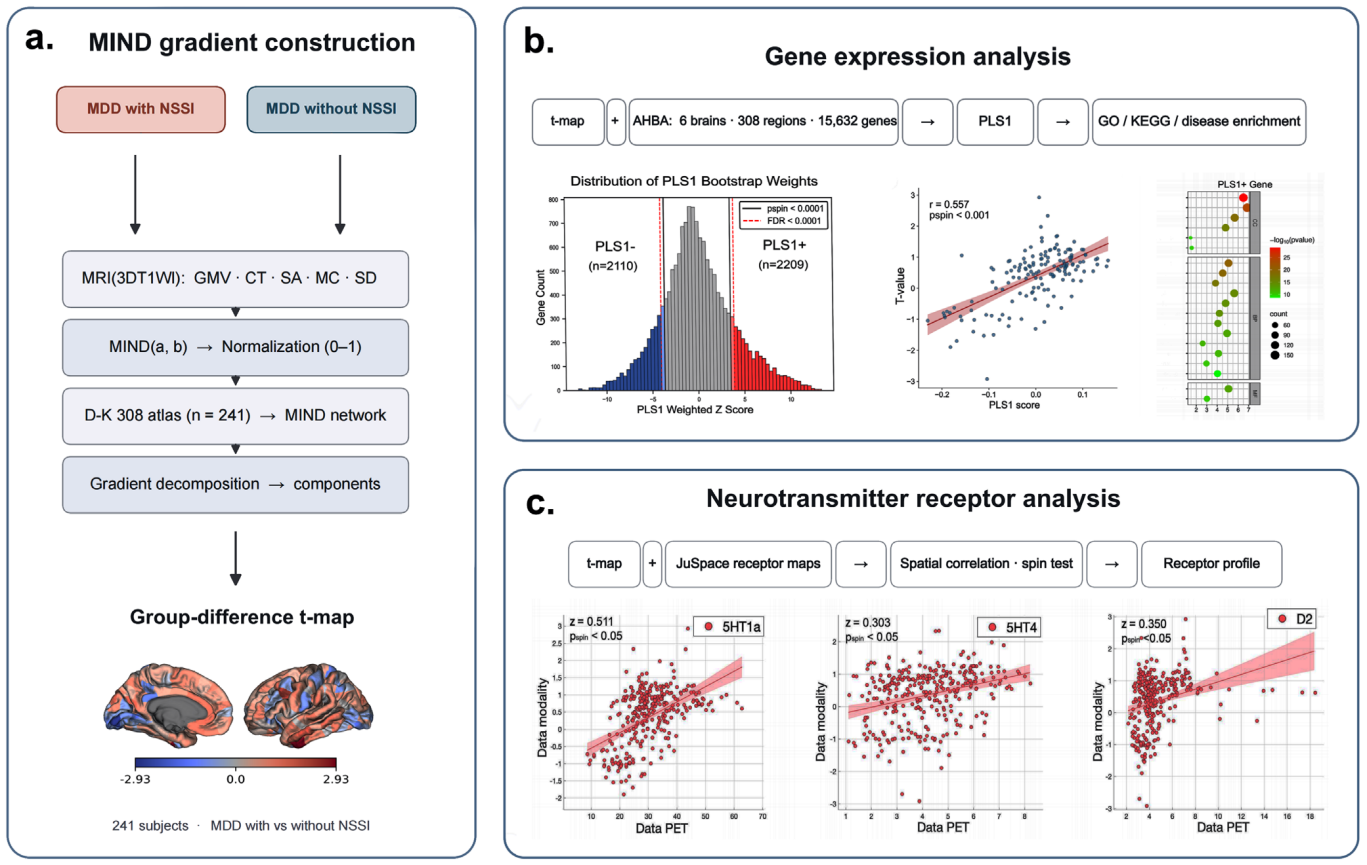
Workflow of this study. (a) MIND gradient construction. Regional morphological features, including gray matter volume (GMV), cortical thickness (CT), surface area (SA), mean curvature (MC), and sulcal depth (SD), were used to construct the Morphometric INverse Divergence (MIND) similarity network within the D‐K 308 atlas. After normalization, diffusion embedding was applied to derive the gradient components, and the group‐difference *t*‐map between NSSI group and non‐NSSI group was obtained. (b) Gene expression analysis. The group‐difference *t*‐map was combined with gene expression profiles from the Allen Human Brain Atlas (AHBA) in a partial least squares (PLS) regression, and the resulting PLS‐weighted genes were further analyzed by Gene Ontology (GO), Kyoto Encyclopedia of Genes and Genomes (KEGG), and disease‐related enrichment, and so forth. (c) Neurotransmitter receptor analysis. Spatial correlations between the group‐difference *t*‐map and JuSpace receptor maps were assessed with spin‐permutation tests, yielding the receptor profile associated with the MIND gradient alterations.

## 2 Methods

### 2.1 Participants

This study retrospectively included 3D T1‐weighted magnetic resonance imaging (3D T1WI) data from 241 adolescent patients with MDD diagnosed according to ICD‐10 criteria at the Shandong Mental Health Center. Based on clinical assessments of NSSI, patients were divided into an NSSI group (134 cases) and non‐NSSI group (107 cases). NSSI was determined based on clinical history documentation and physical examination findings. We reviewed medical records for documented evidence of deliberate self‐inflicted damage to body tissue visible on physical examination, occurring on at least five occasions within the past 12 months and summarized in Sheet S1. The severity of depressive symptoms was quantified using the 17‐item Hamilton Depression Rating Scale (HDRS‐17), with a baseline score of ≥ 18 required for inclusion. All patients were of Han ethnicity and showed no clear lesions or developmental abnormalities on MRI scans (see Sheet S1 for details). As this study is a retrospective analysis and all data were obtained from an imaging database, informed consent was waived. The study was reviewed and approved by the Ethics Committee of the Shandong Mental Health Center (Approval Number: KYSJWLL2024‐1‐075).

### 2.2 Imaging Acquisition and Preprocessing

Imaging data were collected using a 3.0 T uMR 790 MRI scanner (United Imaging Healthcare, China). High‐resolution T1‐weighted anatomical images were acquired with a 3D gradient‐recalled echo sequence (GRE‐FSP). Specific imaging parameters are provided in Table S1. Surface‐based preprocessing of 3D T1 images was performed using FreeSurfer software (v7.4.1, http://surfer.nmr.mgh.harvard.edu/) (Fischl 2012). The preprocessing steps included skull stripping, tissue segmentation, division of hemispheres and subcortical structures, and generation of gray‐white matter and pial surfaces. The preprocessed images were initially reviewed by two experienced radiologists with over 10 years of experience. Euler numbers were calculated for each segmented T1‐weighted image, and images with Euler numbers less than −270 were excluded (see Supporting Information: Text). Total intracranial volume (TIV) was also calculated (Rosen et al. 2018; Monereo‐Sánchez et al. 2021).

### 2.3 Construction of the MIND‐Gd

The cortical surface was divided into 308 spatially continuous regions (D‐K 308 atlas) (Desikan et al. 2006; Romero‐Garcia et al. 2012) by segmenting 68 cortical regions from the Desikan‐Killiany (D‐K) atlas. A recursive algorithm was used to ensure that each region had a similar surface area (approximately 500 mm^2^). The segmentation results were then individually mapped onto the cortical surface of each participant. Five morphological features were extracted: gray matter volume (GMV), cortical thickness (CT), surface area (SA), mean curvature (MC), and sulcal depth (SD) (Sebenius et al. 2023). These features were standardized using z‐score normalization. Next, transformed Kullback–Leibler (KL) divergence was used to calculate MIND similarity metrics between any two cortical regions, resulting in a 308 × 308 MIND similarity matrix for each participant (see Figure 1, Supporting Information: Text and Figure S1). The BrainSpace toolbox was then used to preprocess the MIND statistical gradients (Vos De Wael et al. 2020). Since the principal gradient is closely related to basic cortical properties and functional mapping (Wagstyl et al. 2020), this study focused on the principal morphological statistical gradient (*n*_components = 1) and its changes associated with NSSI in MDD. Finally, a group‐level matrix was generated by averaging individual matrices. For individual‐level gradient estimation, individual affinity matrices were aligned to the group‐level embedding using Procrustes rotation to resolve sign ambiguity as implemented in the BrainSpace toolbox (see Supporting Information: Text and Table S2).

### 2.4 Analysis of Regional MIND‐Gd

To explore differences in the principal MIND gradient across 308 cortical nodes between groups, we performed regression analysis using a general linear model (GLM) with age, sex, age × sex, and TIV as covariates. Two‐tailed *t*‐tests were then conducted with cluster‐level false discovery rate (Cluster‐FDR) correction (*p* < 0.05; see Figure S2). To further clarify changes within broader cortical classification frameworks, all 308 cortical regions were mapped onto two classic schemes: the Yeo‐7 resting‐state network atlas (Thomas Yeo et al. 2011) and the von Economo cytoarchitectonic atlas (Von Economo and Koskinas 2008). Mean principal MIND gradients were calculated for all regions within each network and cytoarchitectonic class. Subsequently, GLM models analyzed MIND gradient differences between groups while controlling for the same covariates, with uncorrected *p* < 0.05, labeled as exploratory.

### 2.5 Association of Neurotransmitter Receptors With MIND‐Gd

To elucidate molecular mechanisms underlying NSSI‐related changes in regional MIND‐Gd, we examined spatial correlations between group‐difference *t*‐maps and neurotransmitter receptor distribution maps derived from PET imaging datasets provided by the JuSpace toolbox (Dukart et al. 2021). Neurotransmitter maps were first mapped onto the DK‐308 brain atlas. Spatial correlations were then calculated between group‐difference *t*‐maps and each neurotransmitter receptor map. Significance was assessed through 10,000 spin tests with Bonferroni correction (*p*_spin_ < 0.05; see Supporting Information: Text and Table S3).

### 2.6 Gene Expressional Profiles Preprocessing

Gene expression profile data were obtained from postmortem brain tissue samples of six individuals, covering 3702 spatially distributed gene loci from the AHBA database (http://human.brain‐map.org) (Hawrylycz et al. 2012; Arnatkeviciute et al. 2023). Since only two AHBA samples included right hemisphere data, this study focused exclusively on the left hemisphere. The AHBA dataset was preprocessed using the Python‐based abagen toolbox (https://github.com/rmarkello/abagen). Gene expression profiles were mapped onto 152 brain regions of the left hemisphere in the DK‐308 atlas, resulting in a transcriptional expression matrix of 152 brain regions × 15,632 genes (Markello et al. 2021).

### 2.7 Transcription‐Neuroimaging Association Region PLS Analysis

To associate gene expression profiles with regional MIND‐Gd changes, partial least squares (PLS) regression was performed. Expression levels of 15,632 genes across 152 brain regions were predictors, and group‐difference *t* values of 152 MIND‐Gd regions were response variables (Krishnan et al. 2011; Abdi and Williams 2013). Spatial autocorrelation with 10,000 permutations assessed whether PLS‐explained covariance exceeded chance. Given no consensus on the optimal component, PLS1 or PLS2 is commonly used. We selected components with variance explained > 30% and significance (*p*_spin_ < 0.0001) (Mao et al. 2024). Gene significance was evaluated via 10,000 bootstrap iterations; *Z* values were expression weights divided by bootstrap standard errors and ranked by weight (Li et al. 2021; Váša et al. 2018). Only BH‐FDR corrected *p* < 0.0001 genes were retained. Spearman correlation examined spatial relationships between MIND‐Gd t‐maps and PLS scores, validated via 10,000 permutations. For NSSI exploration, genes from prior studies (Drevets et al. 2022; Chawla et al. 2025) were screened. Neurotransmitter‐related genes overlapping with significant PLS+ genes were analyzed for spatial correlation with the group‐difference t‐map. From significant PLS1+ genes, NSSI candidates were identified by (1) intersecting with a published NSSI gene list; (2) retaining genes with significant spatial correlation to the group‐difference *t*‐map (*p*_spin_ < 0.0001, BH‐FDR corrected).

### 2.8 Enrichment Analysis

To analyze disease‐related pathways associated with significant PLS+/− genes, we selected statistically significant genes from the first PLS component (PLS1+/−) and performed functional annotation using Gene Ontology (GO) and Kyoto Encyclopedia of Genes and Genomes (KEGG) pathway databases on the Metascape platform (https://metascape.org/gp/index.html#/main/step1) (Zhou et al. 2019). Cortical layer enrichment analysis was conducted using marker genes for six cortical regions from published transcriptomic studies (He et al. 2017). Cell‐type specificity was validated by comparing significant PLS1+/− gene lists with gene sets from seven cortical cell types, including microglia, oligodendrocytes, endothelial cells, astrocytes, oligodendrocyte precursor cells (OPCs), excitatory neurons, and inhibitory neurons (Li et al. 2021). GO and KEGG enrichment analyses were Bonferroni‐corrected (*p* < 0.05), while cortical layer and cell‐type specificity analyses were corrected using BH‐FDR (*p* < 0.05). All enrichment analyses were evaluated using 10,000 spin tests.

### 2.9 Null Model

To address potential confounding effects caused by spatial autocorrelation (Váša et al. 2018; Alexander‐Bloch et al. 2018), we used spin test null models. This method generates Pearson correlation coefficients under the null hypothesis by randomly rotating spherical projections of spatial maps while preserving spatial topology. Specifically, 10,000 spin permutations were applied to cortical regions to construct a null distribution. The pspin value was calculated as the proportion of null correlation coefficients exceeding the observed value.

### 2.10 Robustness Analysis

To ensure reliability and robustness, we conducted five additional analyses: (a) verifying robustness of group‐difference MIND gradients by regressing out TIV; (b) validating MIND‐Gd network construction using Spearman correlation; (c) assessing cluster size thresholds (*n* = 5, 10, 15, 20) on network topology; (d) randomly dividing NSSI patients into two subgroups and comparing MIND‐Gd differences between each subgroup and the non‐NSSI group; and (e) evaluating enrichment robustness using gene category enrichment analysis (GCEA) based on integrated null models (Liu et al. 2020).

## 3 Results

### 3.1 Data Samples and Characteristics

This study included 241 adolescent participants (mean age: 15.91 ± 2.15) who passed data quality control. Among them, 134 patients had MDD with NSSI (mean age: 15.41 ± 1.98), and 107 patients had MDD without NSSI (mean age: 16.53 ± 2.21). Details about image quality control, demographic information, clinical data, and global data residual analysis are provided in Figure S3 and Tables S4 and S5. The mean residuals were close to zero, which confirmed that the model was unbiased.

### 3.2 Group‐Difference Differences in MIND‐Gd

In the regional principal MIND gradient, areas with similar connectivity patterns are shown in the same color. As illustrated in Figure 2a, the variance explained by the principal MIND gradient was generally similar between groups. Both groups exhibited higher values in the occipital cortex and lower values in the frontal and temporoparietal cortices, reflecting the hierarchical organization of brain tissues. After controlling for age, sex, and age × sex as covariates, the average distribution of the principal MIND gradient differed significantly between groups (two‐sample Kolmogorov–Smirnov test, *p* = 1.11 × 10^−4^, Figure 2b; details in Table S6). The group‐difference *t*‐map showed that positive *t* values indicate an increase in NSSI patients, while negative *t* values indicate a decrease. Regional comparison revealed that NSSI patients showed increased gradient in the left caudal middle frontal (Part 4), left inferior temporal (Part 2), left middle temporal (Part 2), and right inferior temporal (Part 2) regions, and decreased gradient in the left lingual (Part 3) and right superior parietal (Part 1) regions (Figure 2c and Table S7). Additionally, a significant negative correlation was found between the average regional MIND gradient in non‐NSSI patients and the group‐difference *t*‐map (*r* = −0.708, *p* < 0.01, Figure 2d), suggesting that highly connected regions show larger group differences, particularly at extreme gradient values.

**FIGURE 2:**
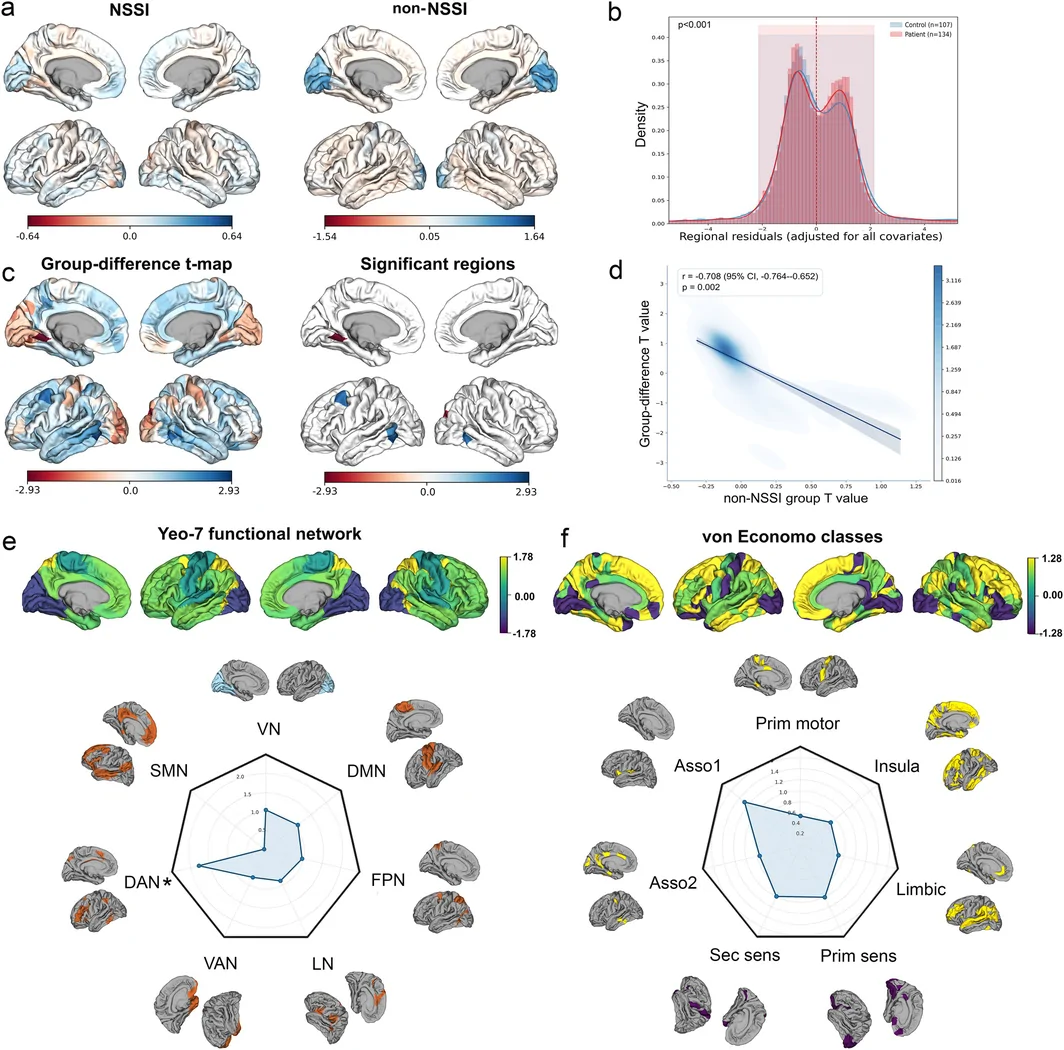
NSSI‐related regional changes in principal MIND gradient map. (a) The principal MIND gradient pattern in MDD patients with NSSI and non‐NSSI. Regions with similar connectivity patterns show similar colors. And the variance explained was generally similar between the NSSI and non‐NSSI groups: both groups exhibited higher values in the occipital cortex and lower values in the frontal and temporoparietal cortices. (b) The histogram shows the distributions of principal MIND gradient in the NSSI and non‐NSSI group after regressing out the effect of age, sex, age × sex, and TIV. (c) Region‐wise statistical comparisons NSSI patients showed an increase in the left caudal middle frontal (Part 4), left inferior temporal (Part 2), left middle temporal (Part 2), and right inferior temporal (Part 2) regions. In contrast, a decrease was observed in the left lingual (Part 3) and right superior parietal (Part 1) regions. (d) The scatterplot of the mean control and group‐difference *t*‐map. The group‐difference *t* values exhibited a negative spatial correlation with the regional MIND gradient values (*r* = −0.708, *p*_spin_ = 0.002). (e) Functional community‐based absolute *t* value (left, Yeo‐7 functional networks) and cytoarchitecture‐based absolute *t* value (right, von Economo classes) of the principal MIND gradient. Left showed a marginally significant decrease in the DAN (*p* = 0.07). * indicate marginally significant (*p* = 0.05–0.10). Asso1, association cortex1; Asso2, association cortex2; DAN, dorsal attention network; DMN, default mode network; FPN, fronto‐parietal network; Insula, insular cortex; Limbic, limbic regions; LN, limbic network; Prim motor, primary motor cortex; Prim sens, primary sensory cortex; Sec sens, second sensory cortex; SMN, somato‐motor network; VAN, ventral attention network; VN, visual network.

To further interpret the group differences, we applied two cortical parcellation methods: the Yeo‐7 functional network atlas and the von Economo cytoarchitectonic atlas. In the Yeo functional network, MDD patients with NSSI showed a marginally significant decrease in the principal MIND gradient within the dorsal attention network compared to the non‐NSSI group (Figure 2e, Figure S4, Table S8). In the von Economo atlas, no significant changes were observed between groups (Figure 2f, Figure S4, Table S9).

### 3.3 Association of Neurotransmitter Receptor Systems With MIND‐Gd Alterations

We examined the association between regional MIND‐Gd changes and whole‐brain cortical neurotransmitter receptor maps. Among the 30 neurotransmitter maps provided by JuSpace, we observed that the group‐difference t‐map showed significant spatial correlations with nine neurotransmitters (Figure 3 and Sheet S2).

**FIGURE 3:**
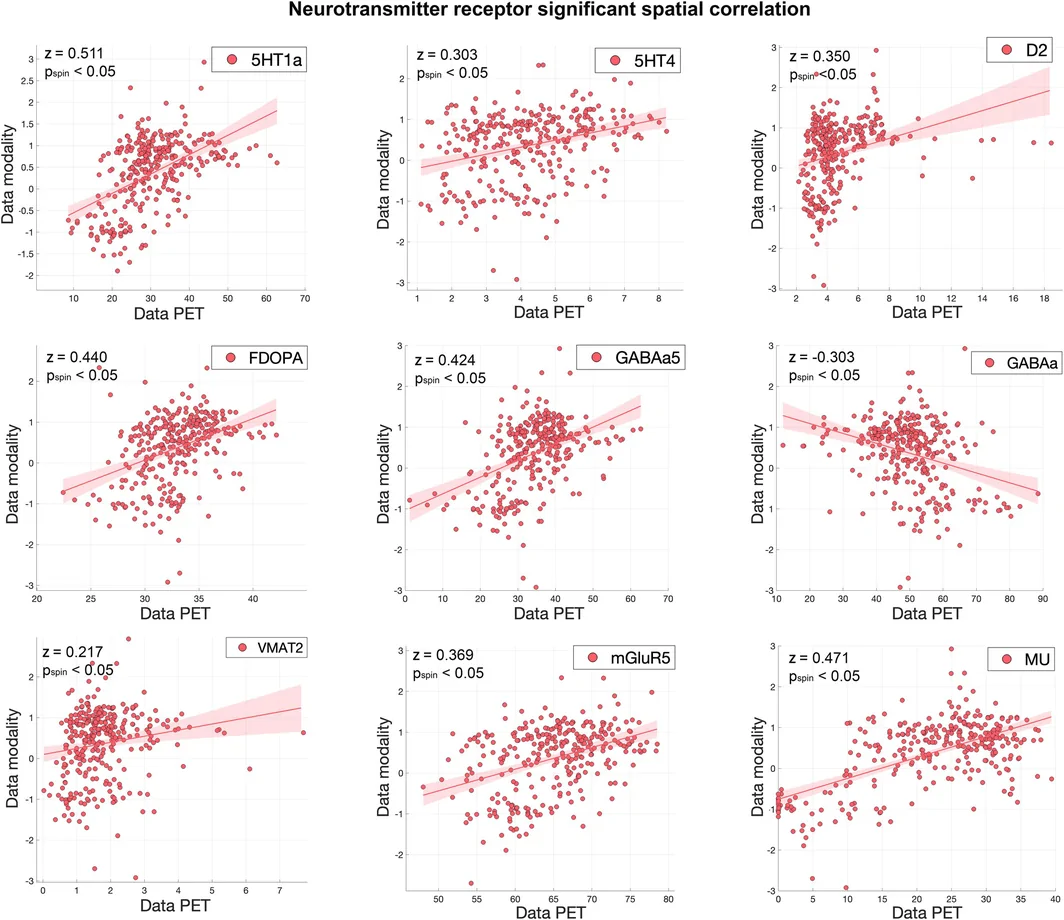
Significant spatially correlated neurotransmitter scatter plot. The above nine neurotransmitters were analyzed using Spearman correlation analysis and subjected to 10,000 permutation tests. The scatter plots of neurotransmitters with statistical significance (*p*_spin_ < 0.05, Bonferroni‐corrected) were displayed with a 95% confidence interval range.

### 3.4 Transcription‐Neuroimaging Associations

Partial least squares regression (PLS) revealed that PLS Component 1 explained 39.53% of macroscopic structural variance, significantly higher than random expectations (*p*_spin_ < 0.0001). PLS1 contributed 73.12% of the variance (Sheet S3 and Figure S5). The weighted gene expression map of PLS1 increased progressively from the precentral gyrus to the inferior temporal gyrus (Figure 4a). The PLS1 score showed significant positive spatial correlation with the group‐difference *t*‐map (*r* = 0.557, *p*_spin_ < 0.001, Figure 4b), indicating that PLS1‐contributing genes were overexpressed in regions with increased gradient in NSSI patients. Using z‐scores, we identified 2209 PLS1+ genes (*Z* > 4.19, BH‐FDR corrected *p* < 0.0001) and 2110 PLS1− genes (*Z* < −4.19, BH‐FDR corrected *p* < 0.0001) (Figure 4c, Sheet S4). Since NSSI‐related gene databases are limited, we combined findings from previous studies (Fulcher et al. 2021; Otte et al. 2016). From the PLS1+ gene list, we identified two genes closely related to NSSI in MDD: GABRA5 and MGST1. Both showed significant spatial correlations with the group‐difference *t*‐map (*p*_spin_ < 0.0001, BH‐FDR corrected, Figure 4d,e).

**FIGURE 4:**
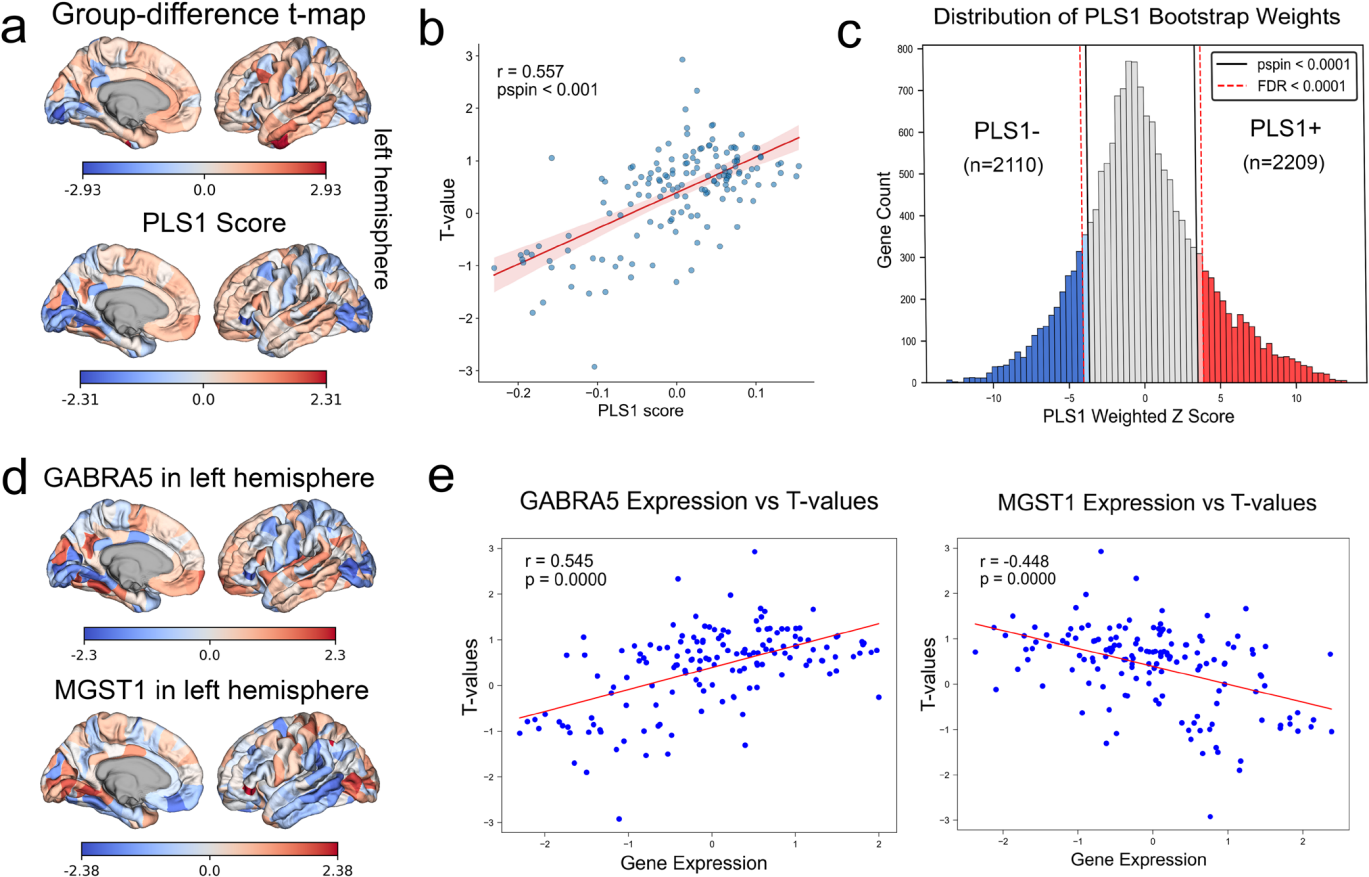
Transcriptional signatures associated with group‐difference regional changes in principal MIND gradient and two prominent genes. (a) The group‐difference *t*‐map of the regionally principal MIND gradient scores in the left hemisphere and the weighted gene expression map of regional PLS1 scores in the left hemisphere. The two maps have similar distributions, both showing a gradually increasing pattern from the parietal lobe to the frontotemporal lobe. (b) The scatterplot showing significant positive spatial correlations between PLS1 scores and the group‐difference *t* value maps in the principal MIND gradient (Spearman's *r* = 0.557, *p*_spin_ < 0.001). (c) A total of 2209 PLS1+ genes (*Z* > 4.19, *p*_FDR < 0.0001) and 2110 PLS2− genes (*Z* < −4.19, *p*_FDR < 0.0001) were identified by ranked *Z* scores. (d–e) GABRA5 and MGST1 gene expression maps in the left hemisphere reveal distinctly opposing distribution patterns. The scatterplot demonstrates a significant positive spatial correlation between GABRA5 expression and the group‐difference *t* value maps within the principal MIND gradient (Spearman's *r* = 0.545, *p* < 0.0001), alongside a significant negative spatial correlation for MGST1 expression (Spearman's *r* = −0.448, *p* < 0.0001).

### 3.5 Annotation of PLS‐Weighted Genes for Regional MIND‐Gd Changes

Functional enrichment analysis using Metascape revealed that PLS1+ genes were significantly enriched in synaptic signaling and neurodevelopment‐related pathways (Figure 5a,b), whereas PLS1− genes were predominantly associated with chromatin regulation, cytoskeletal organization, and nucleic acid metabolism (Figure 5c,d). When aligning human diseases with the PLS1 genes, we found that the PLS1+ genes were significantly enriched in neurological and psychiatric disorders such as depression and epilepsy, whereas the PLS1‐ genes were enriched in neurodevelopmental disorders including autism and developmental delay (Figure 5e,f).

**FIGURE 5:**
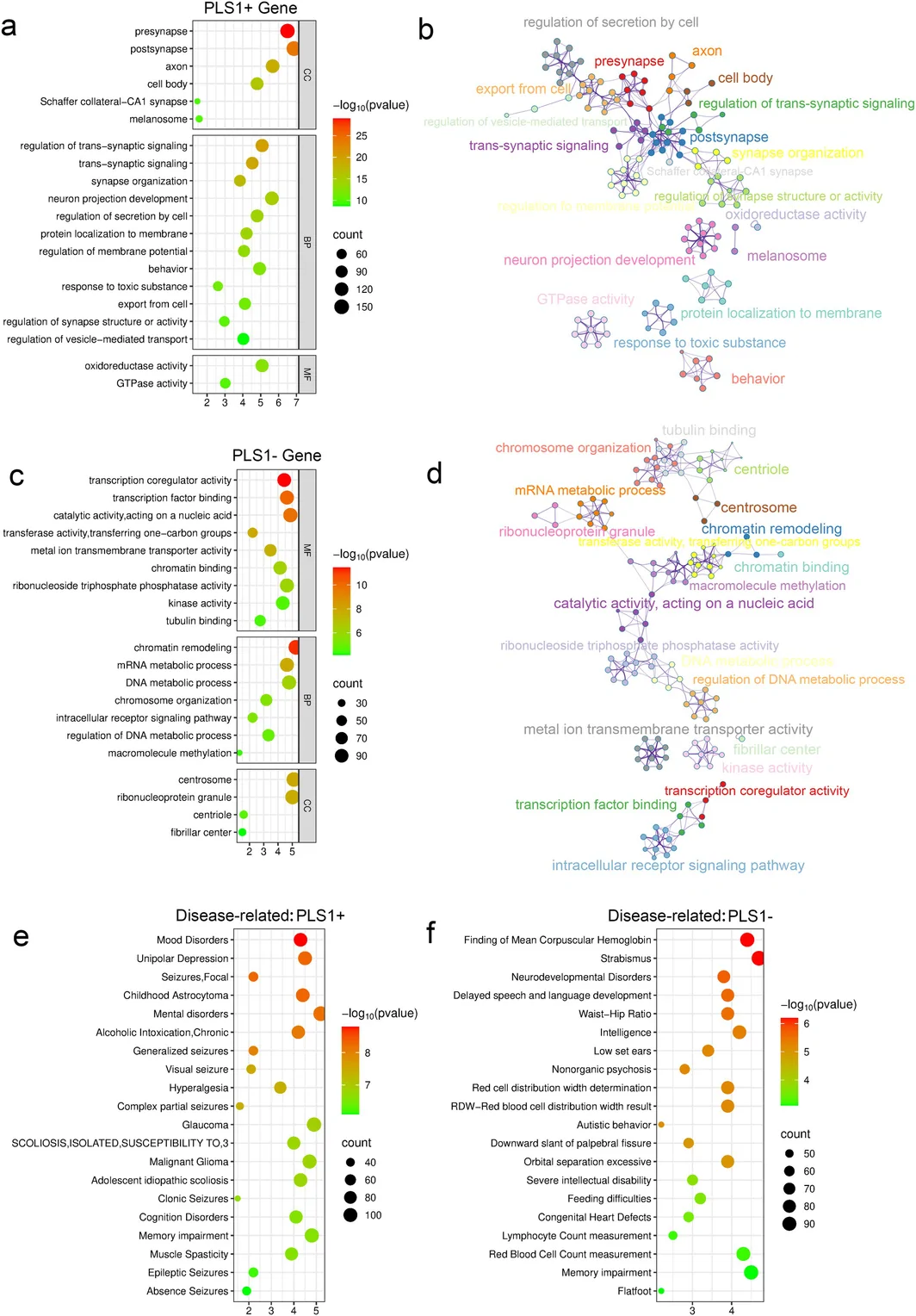
Functional enrichment analysis of PLS1 weighted genes. (a and c) The bubble diagram showing the enrichment of GO terms and pathway annotation for the PLS1+/− genes. The *x*‐axis represents −log10(*p* value) corrected by BH‐FDR method. The size of bubble represents the number of genes included in each GO term and pathway. (b and d) Metascape analysis showed the enrichment network comprised of inter‐cluster and intra‐cluster similarities of enriched terms and pathways for PLS1+/− (d) genes. The node represents a term or pathway, and the size of node represents the number of genes included in each GO term and pathway. Lines among nodes represent links among terms and pathways, and colors represent different clusters. (e and f) The bubble diagram shows the enrichment of disease‐related pathways associated with the PLS1+/− genes. The *x*‐axis represents the −log10(*p* value) corrected by the BH‐FDR method, and the size of each bubble represents the number of disease‐related genes in each pathway. *Source:* DisGeNET.

### 3.6 Cortical Layer Enrichment Related to Regional MIND‐Gd Changes

Using cortical gene markers from previous studies, we established the relationship between PLS1+/− genes and different cortical layers (L1–L6) through laminar gene marker analysis (Sheet S5). Notably, PLS1+ genes were significantly enriched in Cortical Layer I (gene overlap number = 87, BH‐FDR corrected *p* = 0.015), Layer II (gene overlap number = 140, BH‐FDR corrected *p* < 0.001), and Layer V (gene overlap number = 30, BH‐FDR corrected *p* < 0.001). In contrast, PLS1− genes were significantly enriched in Layer III (gene overlap number = 63, BH‐FDR corrected *p* < 0.001) and Layer IV (gene overlap number = 83, BH‐FDR corrected *p* < 0.001). There was no overlap in the enrichment of PLS1+/− genes across different cortical layers (Figure 6a,b).

**FIGURE 6:**
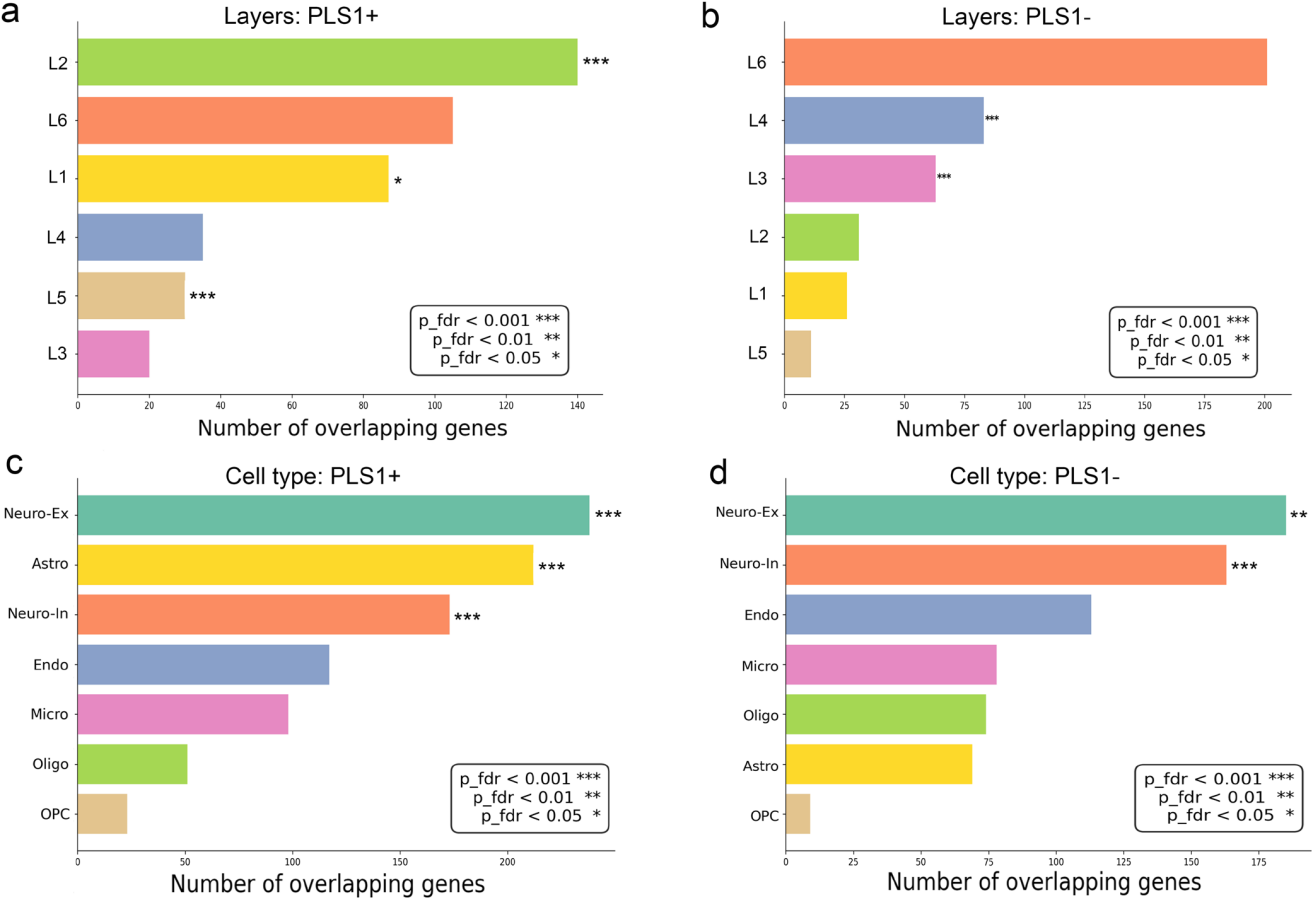
Cortical layer and cell‐type specific enrichment analysis of the PLS1+/− gene list. (a and b) The number of overlapped genes with PLS1+/− genes for each cortical layer. The PLS1+ gene list was significantly enriched in Cortical Layers I, II, and V, while the PLS1− gene list was significantly enriched in Layers III and IV. There was no overlap in the enrichment regions between the two gene sets. (c and d) The number of overlapped genes with PLS1+/− genes for each cell‐type. PLS1+/− genes were enriched in both excitatory and inhibitory neurons, but an increase in PLS1+ genes was significantly associated with astrocytes.

### 3.7 Specific Cell Types Enrichment Related to Regional MIND‐Gd Changes

Given the complex interactions among different cell types in the brains of adolescents with MDD and NSSI, we further explored the cell‐specific changes in the principal MIND gradient. To refine our analysis, we included seven types of central nervous system cells (Sheet S6). The cell‐type‐specific expression analysis across seven CNS cell types revealed that PLS1+ genes were significantly enriched in excitatory neurons (238 overlap genes, BH‐FDR corrected *p* < 0.001), astrocytes (212 overlap genes, *p* < 0.001), and inhibitory neurons (173 overlap genes, *p* < 0.001). PLS1− genes were enriched in excitatory neurons (185 overlap genes, *p* = 0.009) and inhibitory neurons (163 overlap genes, *p* < 0.001). Notably, only PLS1+ genes showed significant association with astrocytes (Figure 6c,d).

### 3.8 Sensitivity and Robustness Analyses

Sensitivity analyses confirmed the robustness of our findings: (a) group differences were not sensitive to TIV (*r* = 1.000, *p*_spin_ < 0.001, Figure S6); (b) Spearman‐based MIND‐Gd showed high reproducibility (*r* = 0.999, *p*_spin_ < 0.001, Figure S7); (c) cluster size thresholds (*n* = 5,10,15,20) did not affect topological metrics (*p* > 0.05, Table S10 and Figure S8); (d) dividing patients with NSSI into two random subgroups (Subgroup 1 and Subgroup 2) and assessing their MIND‐Gd differences compared to the non‐NSSI group. The group‐difference *t*‐maps showed positive spatial correlations with Subgroup 1 (*r* = 0.912, *p*_spin_ = 0.002) and Subgroup 2 (*r* = 0.933, *p*_spin_ < 0.001, Figure S9); (e) relatively reliable gene enrichment results (Supporting Information: Text and Sheet S7).

## 4 Discussion

To date, no studies have specifically investigated brain structural and network changes in adolescent MDD patients with or without NSSI. This study is the first to systematically explore these differences and their potential molecular mechanisms using an integrated analytical approach. By constructing cortical structural similarity gradients based on the principal MIND gradient network, we identified clear group differences between the two groups, revealing distinct alterations in neurotransmitter activity and structural connectivity among NSSI patients. Using partial least squares (PLS) analysis, we found that MIND‐Gd differences were significantly correlated with cortical gene expression maps. We also identified two key genes, GABRA5 and MGST1, that may play pivotal roles in NSSI pathophysiology. Gene enrichment analysis showed that PLS1+ genes were enriched in synaptic signaling pathways, whereas PLS1− genes were enriched in chromatin regulation and cytoskeletal processes. Cortical laminar analysis revealed that PLS1+ genes were enriched in Layers I, II, and V, while PLS1− genes were enriched in Layers III and IV. Cell‐type analysis further showed significant enrichment of PLS1+ genes in astrocytes. These findings highlight abnormal MIND‐Gd phenotypes in adolescent MDD patients with NSSI and provide new insights into the relationship between large‐scale structural abnormalities and microscopic transcriptional patterns.

MIND is more reliable, better aligned with cellular structure, and more sensitive in detecting individual brain connectivity differences (Sebenius et al. 2023). Furthermore, it is closely linked to fundamental cortical properties such as gene expression, cellular structure, myelin architecture, and evolutionary expansion (Havinga et al. 2017; Monereo‐Sánchez et al. 2021). Adolescent MDD, whether accompanied by NSSI or not, disrupts neuroplasticity through multiple factors including genetics, gene–environment interactions, neuroendocrine dysfunction, and inflammation (Otte et al. 2016; Liu, Guan, et al. 2024). To capture the subtle differences, we leveraged the advantages of the MIND network to construct a principal MIND gradient model with enhanced sensitivity to cortical hierarchical organization. Our results revealed region‐specific alterations: the gradient increased in frontal and temporal regions but decreased in parietal and occipital regions. Increased gradients suggest reduced structural differentiation, while decreased gradients indicate greater structural segregation. In these regions, the left occipital area processes initial visual information, bilateral temporal pathways are involved in visual memory and object recognition, the right parietal region integrates spatial attention and somatosensory information, and the left frontal region regulates attention, decision‐making, and executive functions (Kang et al. 2020; Yomogida et al. 2017; Patil et al. 2021). The visual functional system appears to play a key role, supporting the hypothesis that visual dysfunction may be an important clinical feature of MDD (Lu et al. 2020). Additionally, NSSI might be linked to immature rational decision‐making during adolescence, which, combined with limbic hyperactivity, could lead to emotional dysregulation triggered by visual perception and memory (Plener et al. 2012; Huang et al. 2021; Zhou et al. 2022). We also identified attention‐related mechanisms: the right superior parietal lobule, a core node of the dorsal attention network (DAN), works with the left caudal middle frontal gyrus and left middle temporal gyrus to regulate goal‐directed attention. The marginal significance of DAN network edges in our study further supports this finding. These findings suggest that adolescents with NSSI may exhibit compensatory responses in cognitive processing before adulthood, closely related to the unique physiological, psychological, and social changes of adolescence (Wang et al. 2025; Niu et al. 2023). Finally, reproducibility analyses confirmed that group differences were not influenced by TIV, correlation methods, or cluster size thresholds.

We examined neurotransmitter receptor distributions along the principal MIND gradient. The group‐difference *t*‐map was spatially correlated with nine receptor maps from normative adult PET templates. These receptors primarily signal through GPCR pathways involved in synaptic plasticity, emotional regulation, impulse control, and pain‐reward processing. Specifically, serotonergic and GABAergic systems were spatially linked to emotional regulation and impulse control (Wood et al. 2022; Deligiannidis and Clayton 2023), while mGluR5 was related to emotional regulation (Davis et al. 2019). Dopamine and opioid systems were associated with pain‐reward reinforcement mechanisms (Xu et al. 2023; Störkel et al. 2021; Bresin and Gordon 2013), consistent with prior NSSI literature (Mantas et al. 2022; Monfared et al. 2021). These spatial correlations indicate alignment between NSSI‐related structural gradient differences and regions of high receptor density, but must be interpreted cautiously as associations with normative adult distributions rather than direct evidence of altered receptor activity in our adolescent sample.

Pathway enrichment analysis using PLS revealed key transcriptional features. In the PLS1+ gene set, synapse‐related functions were significantly enriched, including “glutamatergic synapse” (GO:0060079), “chemical synaptic transmission” (GO:0007268), and “regulation of trans‐synaptic signaling” (GO:0099177). Key genes such as GABRA5, SYT17, KCNMB4, EFNB3, and SSTR1 are involved in synaptic receptor function, vesicle release, and signal balance (Romero‐Garcia et al. 2019; Zhu et al. 2016; Brenner et al. 2005). The PLS1+ gene set also contributes to neural development; for example, DPYSL3 is involved in axon guidance and works with cytoskeletal regulators like TMSB10 and IFT22 to regulate neuronal projection growth and direction (Van Der Ende et al. 2023; Zhao et al. 2025; Sasayama et al. 2013). This gene set is linked to mood disorders, epilepsy, and cognitive impairments, suggesting it may influence NSSI mechanisms through a “synapse‐development‐behavior” network. In contrast, the PLS1− gene set was associated with transcriptional and epigenetic regulation, enriched in pathways such as “transcription coregulator activity” (GO:0003712), “chromatin remodeling” (GO:0006338), and “chromatin binding” (GO:0003682). Core genes include CUX1, NCOA3, and RORA, which regulate transcription and chromatin accessibility (Rybczyński et al. 2026; Logue et al. 2013; Szklarczyk et al. 2021). PLS1− genes were also enriched in “centrosome” (GO:0005813), “microtubule binding” (GO:0015631), and “DNA metabolic process” (GO:0006259), with genes such as TUBD1, CEP95, EEPD1, and AGO1 involved in structural maintenance and metabolism (Liu et al. 2022; Nickoloff et al. 2022; Niu et al. 2022). Phenotypic analysis linked PLS1− genes to neurodevelopmental disorders including intellectual delay, autism‐related behaviors, and morphological abnormalities. Thus, PLS1+ and PLS1− gene sets have distinct roles: the former in synaptic and developmental regulation, the latter in basic cellular processes. We also identified GABRA5 and MGST1 as overlapping with neurotransmitter‐related analysis. Variations in GABRA5 significantly increase NSSI genetic risk, possibly by altering temporal lobe gray matter volume and disrupting social–emotional networks (Sun et al. 2025). MGST1 regulates B‐cell activation and cytokine release such as IL‐6, closely related to the inflammatory hypothesis of MDD (Morabito et al. 2021).

Through combined cortical layer and single‐cell expression analyses, we found that PLS1+/− genes are differentially enriched across cortical layers. Both gene sets are enriched in excitatory and inhibitory neurons, implying they may contribute to fine‐tuning synaptic transmission and network oscillations critical for cognitive flexibility and emotional regulation. However, PLS1+ also shows significant expression in astrocytes, highlighting their regulatory role in maintaining neuronal and synaptic physiology during brain homeostasis. Combined with MGST1 findings, this supports the inflammation hypothesis of MDD, which suggests that astrocyte‐released cytokines mediate neuroinflammation (González‐Arias et al. 2023; Zhao et al. 2022).

Overall, our results suggest that neurons, astrocytes, and their connectivity patterns provide critical neurobiological insights into adolescent MDD with NSSI. Early intervention during this vulnerable period may mitigate long‐term disease progression and reduce psychiatric outcomes. Future research with larger and independent samples is needed to determine whether these neurobiological signatures may inform our understanding of disease mechanisms and intervention targets.

Despite its significant contributions, several limitations warrant consideration. First, the MIND network was built using T1 images and five morphological features (Yang et al. 2021; King and Wood 2020); future studies should incorporate larger‐scale microstructural indices and multimodal imaging. Second, the relatively small sample size limits generalizability and assessment of individual differences. Third, as a retrospective study, we could not systematically collect data on clinically important variables known to influence NSSI, including suicidal ideation, impulsivity, childhood trauma, anxiety symptoms, and medication exposure. Undetected confounding is therefore possible. Fourth, the female predominance in our sample is expected given the higher prevalence of MDD and NSSI in female adolescents, but may limit generalizability to male patients. Sex‐stratified analyses showed high consistency between females and the entire sample (*r* = 0.793, *p*_spin_ = 0.003) and moderate consistency for males (*r* = 0.374, *p*_spin_ = 0.008; Figure S10); however, the smaller male subgroup highlights the need for larger, sex‐balanced cohorts. Fifth, the AHBA dataset consists of postmortem tissue from only six adult donors (mean age ~50 years, predominantly Caucasian), introducing an approximately 35‐year age gap and ethnic disparity relative to our adolescent Han Chinese sample (Mogil et al. 2018), with only left‐hemisphere data available. Gene expression findings should therefore be considered exploratory spatial associations with adult normative reference data. Leave‐one‐donor‐out sensitivity analyses supported PLS1 robustness (*r* = 0.54–0.56, all *p*_spin_ < 0.01; Figure S11). Future studies should use whole‐brain transcriptomic datasets matched for age and ethnicity, and independent replication in larger, prospective cohorts is warranted.

## 5 Conclusion

In conclusion, our findings reveal significant alterations in the principal MIND gradient between adolescent MDD patients with and without NSSI, characterized by increased gradient values in frontal and temporal regions and decreased values in parietal and occipital regions. These alterations showed spatial correlations with neurotransmitter receptor distributions and enrichment in neurobiologically relevant gene pathways. However, given the cross‐sectional and correlational design, these findings should be considered preliminary and hypothesis‐generating. Independent replication in larger, prospective cohorts with comprehensive clinical phenotyping is necessary before any clinical implications can be inferred.

## Author Contributions

**Yan Zhang:** writing – original draft, writing – review and editing, data curation, methodology, visualization formal analysis, and conceptualization. **Xinyuan Hu:** writing – original draft, data curation, methodology, and formal analysis, visualization. **Wei Wang:** writing – review and editing, formal analysis, visualization, and investigation. **Jing Wang:** writing – review and editing, funding acquisition, software, visualization, methodology, and resources. **Hongying Li:** visualization, funding acquisition, methodology, and resources. **Yumei Wan:** writing – review and editing and investigation. **Jianlei Zhu:** project administration, methodology, and investigation. **Fei Liu:** data curation and validation. **Shiyue Tao:** data curation and formal analysis. **Duanwei Wang:** investigation and resources. **Xiangtao Lin:** supervision, investigation, and conceptualization. **Yingying Zhang:** investigation and resources. **Hongmei Zhang:** writing – review and editing, methodology, resources, methodology, and investigation. **Yuandong Gong:** writing – review and editing, funding acquisition, supervision, formal analysis, resources, investigation, and conceptualization. The authors have reviewed and approved the final manuscript and take full responsibility for its content.

## Funding

This work was supported by the National Natural Science Foundation of China (Grant 62406128), the Shandong Provincial Medical and Health Science and Technology Project (2024) (Grant 202403091056), and the Sichuan VS Mental Health Survey and Early Intervention (2023) (Grant 05).

## Ethics Statement

This study was conducted in accordance with the Declaration of Helsinki. Ethical approval was obtained from the Ethics Committee of the Shandong Mental Health Center (ref: KYSJWLL2024‐1‐075).

## Consent

As this was a retrospective analysis utilizing data from an imaging database, the requirement for informed consent was waived.

## Conflicts of Interest

The authors declare no conflicts of interest.

## Supporting information


**Table S1:** Details of sagittal 3D high‐resolution T1‐weighted images.
**Table S2:** Details setting of the BrainSpace toolbox.
**Table S3:** Details of neurotransmitters in Juspace.
**Table S4:** Demographic and clinical characteristics of the subjects.
**Table S5:** Global residual analysis results.
**Table S6:** GLM model summary.
**Table S7:** Regional MIND gradient differences between NSSI and non‐NSSI.
**Table S8:** The results of the Yeo‐7 network.
**Table S9:** The results of the von Economo network.
**Table S10:** Cluster statistics under different thresholds.
**Table S11:** Regional MIND gradient differences of Group 1 and Group 2.
**Figure S1:** Elaboration of MIND. Firstly, the surface was subdivided into 308 contiguous areas based on the D‐K 308 atlas. Each area was characterized by five structural features: surface area (SA), cortical thickness (CT), mean curvature (MC), gray matter volume (GMV), and sulcal depth (SD). To estimate the similarity between cortical regions, we normalized each MRI feature across all vertices and then summarized all MRI measures across all vertices within each cortical region. Furthermore, the *K*‐nearest neighbor density algorithm was used to compile a paired distance matrix to estimate the KL divergence between each pair of regional multivariate distributions. Finally, the KL divergence (a and b) of Regions a and b was transformed to estimate the MIND similarity between regions. The MIND was bounded between 0 and 1, with higher values indicating greater similarity.
**Figure S2:** Spatial cluster connectivity rule.
**Figure S3:** Euler‐number box plot between two groups. Left hemisphere: Group 1: mean: −46.953, standard deviation: 20.356, median: −44.000. Group 2: mean: −46.881, standard deviation: 16.660, median: −44.000. The independent sample *t*‐test results showed a *t* value of −0.030 and a *p* value of 0.976, with no significant difference between the two groups (*p* ≥ 0.05). Right hemisphere: Group 1: mean: −41.308, standard deviation: 17.836, median: −38.000. Group 2: mean: −40.985, standard deviation: 14.990, median: −40.000. The independent sample *t*‐test results showed a *t* value of −0.150 and a *p* value of 0.881, with no significant difference between the two groups (*p* ≥ 0.05). Whole brain: Group 1: mean: −88.262, standard deviation: 35.068, median: −82.000. Group 2: mean: −87.866, standard deviation: 28.603, median: −82.000. The independent sample *t*‐test results showed a *t* value of −0.094 and a *p* value of 0.925, with no significant difference between the two groups (*p* ≥ 0.05). Group 1: without NSSI, Group 2: NSSI.
**Figure S4:** Box plot of group comparison across Yeo‐7 network and von Economo network. The above figure shows inter group comparison on the Yeo‐7 network: blue (without NSSI) and red (NSSI) bins have different distributions in different brain networks (such as VN, SMN, and DAN). Although the bins in the without NSSI group are significantly higher than those in the NSSI group, the median is similar in the VN group. Statistical significance: *p* = 0.0756 in DAN, indicating that the difference between groups is close to statistical significance (marginal significance). No significant outliers were observed, and the data distribution is relatively stable. On the von Economo network below, we did not observe statistically significant differences. And no obvious outliers were observed, indicating a relatively stable data distribution.
**Figure S5:** Relative contribution percentage of PLS1 and PLS2. We used a mixed strategy of 10,000 bootstrap samples combined with multiple linear regression to systematically evaluate the significant contribution of PLS1 and PLS2 scores to the group‐difference *t* value. First, generate 10,000 subsets of data through resampling with replacement; Subsequently, a multiple linear regression model was established with group‐difference *t* value as the dependent variable and PLS1 and PLS2 scores as independent variables to obtain their independent contribution coefficients to the *t* value; Finally, based on the coefficient distribution of 10,000 regression results, calculate the confidence interval and *p* value, and determine the contribution proportion according to the percentage of the absolute value of the standardized coefficient to the total absolute value.
**Figure S6:** Validation of TIV effect on group‐difference principal MIND gradient differences. (a) The group‐difference *t*‐map with controlling for TIV. (b) The group‐difference *t*‐map without controlling for TIV. (c) The results of spatial correlation analysis between the principal MS gradient with and without controlling for TIV. *p* value was calculated based on spin test.
**Figure S7:** The effect of different types of correlation analysis on group differences. (A) The group‐difference *t*‐map of the principal MIND gradient based on Spearman's correlation analysis. (B) The group‐difference *t*‐map of the principal MIND gradient based on Pearson's correlation analysis. (C) The results of spatial correlation analysis between the principal MIND gradient based on Spearman's and Pearson's correlation analysis.
**Figure S8:** Evaluating the network topology characteristics under different cluster thresholds size. It was found that the statistical results of group‐difference MIND‐Gd differences remained highly consistent at each threshold (*n* = 5, 10, 15, 20). The clustering distribution patterns, node connection characteristics, and global topology indicators obtained from different threshold settings showed no significant statistical differences.
**Figure S9:** Comparison of principal MIND gradient differences between NSSI subgroups and without NSSI. (a and c) Group 1 revealed principal MIND gradient differents in the left inferior temporal (Part 2), left lingual (Part 3), right inferior temporal (Part 2), and right superior parietal (Part 1), while Group 2 in the left caudal middle frontal (Part 4), left inferior temporal (Part 2), left lingual (Part 3), right lateral orbitofrontal (Part 4), and right superior parietal (Part 1), as detailed in Table S11. Except for right lateral orbitofrontal region, the remaining brain regions in both groups overlap with those in the entire NSSI group. The overlap rates of differential regions in both two subgroups reach up to 3/5. (b and d) The group‐difference *t*‐map for NSSI patients displayed positive spatial correlations with that of Group 1 (*r* = 0.912, *p*_spin_ = 0.002, spin = 10,000) and Group 2 (*r* = 0.933, *p*_spin_ < 0.001, spin = 10,000).
**Figure S10:** Sex‐stratified analysis of NSSI‐related MIND‐Gd group differences. Node‐wise *t* values from GLM (group‐difference adjusted for age and TIV) in (a) females (*n* = 179; NSSI 110 vs. non‐NSSI 69) and (b) males (*n* = 62; NSSI 24 vs. non‐NSSI 38) plotted against the full‐sample *t*‐map across 308 DK‐308 regions. Female (*r* = 0.793, *p*_spin_ = 0.0032) and male (*r* = 0.374, *p*_spin_ = 0.0078) *t*‐maps were each significantly correlated with the full‐sample pattern (spin test with 10,000 permutations; dashed line, *y* = *x*). The smaller male subgroup warrants caution in interpreting the male pattern.
**Figure S11:** Leave‐one‐donor‐out (LODO) sensitivity analysis for the PLS1‐based transcriptomic association. Bars show the spatial Spearman correlation between PLS1 scores and the group‐difference *t*‐map, recomputed after excluding each of the six Allen Human Brain Atlas (AHBA) donors individually. The dashed red line indicates the full‐model correlation (*r* = 0.557). All LODO models remained significant under 10,000 spatial permutations (all *p*_spin_ < 0.001), with correlations ranging from *r* = 0.539 to 0.563, indicating that no single donor disproportionately drove the observed PLS1 spatial pattern.

**Sheet S1.** Patients general information and Euler‐number.
**Sheet S2**. Results of neurotransmitter receptor.
**Sheet S3**. Explanation of composition differences in PLS1–PLS8.
**Sheet S4**. Results of PLS‐weighted *Z* scores for PLS1+/− genes.
**Sheet S5**. Results of layer enrichment analysis for PLS1+/− genes.
**Sheet S6**. Results of cellular overlapped genes with PLS1+/− genes.
**Sheet S7**. Relatively reliable gene enrichment results.

## Data Availability

The code for calculating the MIND could be found on the github: https://github.com/isebenius/MIND. The toolbox for calculating the Gradient could be found on the github: https://github.com/MICA‐MNI/BrainSpace. The code for PLS analysis could be available at the github: https://github.com/SarahMorgan/Morphometric_Similarity_SZ. The code for the computation of spatial permutation testing could be available at the github: https://github.com/frantisekvasa/rotate_parcellation. Human gene expression data that support the findings of this study are available in the Allen Brain Atlas (human.brain‐map.org/static/download). The codes for gene expression analysis can be found at https://github.com/rmarkello/abagen. The Metascape of gene enrichment analysis is available at http://metascape.org/. Other data will be made available on request.
