# Supplementary material for: Major Depressive Disorder With Non‐Suicidal Self‐Injury in Adolescents: Altered Morphological Inverse Divergence Gradient in Brain Structure and Gene Expression Patterns
Source: Hum Brain Mapp. 2026 Oct 21;47(15):e70653. doi: 10.1002/hbm.70653 (PMC13687784; doi:10.1002/hbm.70653)
Supplement: Supplementary file 1 [file HBM-47-e70653-s002.docx]

**Supplementary material**

**Supplementary text**

1. **Quality control of neuroimaging data**

Visual inspection was performed before image data preprocessing to ensure quality. All images with motion artifacts were excluded. Our two physicians reviewed the processed neuroimaging data. When abnormalities were detected in subjects, we first identified the cause and rerun the preprocessing workflow after corrections. Key issues included poor tissue segmentation and inaccurate removal of non-brain tissue. If newly acquired preprocessed MRI images still showed problems, the subject was excluded.

Then, the Euler number is calculated for the segmented results. In the recon-all process of FreeSurfer, the tessellation step may produce holes and handles, which are saved in the ?h.orig.nofix file. The Euler number is calculated by determining the genus using the formula $x=2-2g$. A higher Euler number (less negative) indicates better quality of FreeSurfer cortical reconstruction data. Since the Euler number varies among different populations (such as adolescents and adults) and across different scanning devices and sequences, there is no fixed threshold. In this study, combining the device and common practices, -270 was chosen as the threshold.

After quality control, the study included 134 MDD patients with NSSI and 107 patients without NSSI, as detailed in Supplementary Sheet S1.

2. **Details of MIND gradient construction**

MIND construction: As input data, we employed high-resolution mesh reconstructions of the cortical surface, generated from T1-weighted MRI scans via FreeSurfer's `recon-all` command. This surface comprises 163,842 vertices per hemisphere within the fsaverage template. Each vertex was characterized by five structural MRI features: CT, SA, Vol, MC and SD. To estimate the similarity between cortical areas, we standardized each MRI feature across all vertices and then aggregated all the MRI metrics for all vertices within each cortical area (defined by D-K 308 atlas) to form a regional multivariate distribution. Next we compiled a pairwise distance matrix using a *k*-nearest neighbor density algorithm to estimate the symmetrized KL divergence(known as Jeffrey’s divergence) between each pair of regional multivariate distributions. Finally, applying the formula $\text{MIND}\text{(}\text{a}\text{,}\text{b}\text{)=}\frac{\text{1}}{\text{1+}\text{KL}\text{(}\text{a}\text{,}\text{b}\text{)}}$, we transformed the KL divergence *KL*(*a*,*b*) for regions *a* and *b* to estimate the inter-areal MIND similarity, bounded between 0 and 1, with higher values indicating greater similarity.

MIND gradient construction: The construction of MIND gradients was preprocessed using the BrainSpace toolbox. we used each 308 × 308 MIND matrix and computed a cosine similarity matrix to capture similarities in structural connectivity profiles. Using diffusion map embedding to characterize the gradient components that explain the variance of the network in descending order. This algorithm can capable of projecting a high-dimensional network matrix into a common low dimensional space. Among them, brain regions with more connections or stronger connections are kept in close proximity, while brain regions with fewer connections or no connections are distributed further away. In this study, we used the default setting of the BrainSpace toolbox, and we followed the recommended manifold parameters, set *α* = 0.5 and *time* = 0, which kept global relations between data points in the embedded space. Next, the MIND gradient template was generated from the average MIND matrix based on both patients with NSSI and without, and the Procrustes rotation was conducted to align the individual’s MIND gradient to the template, as detailed in Supplementary Table S2.

3. **Detailed analysis neurotransmitter receptors by JuSpace**

Firstly, we need to resample the group-difference t-map obtained from GLM analysis of the regional MIND gradient to a resolution of 3mm× 3mm × 3mm. The JuSpace version number we use here is v2.0. Atlas we choose the t map that was resampled before and Study Design we choose 4) set 1 each image (Tests each image from set 1 against a null distribution). Then, we selected all PET/Neurotransmitters in the templates list (as details in Supplementary Table S3) for analysis and set the number of permutations to 10000. Finally, we select the Spearman analysis type and click the Run analysis button to proceed with the analysis.

4. **Validation: gene-category enrichment analysis (GCEA) with ensemble-based null models for Gene Ontology**

This study employed a gene set enrichment analysis framework utilizing a spherical permutation null model generation strategy to assess the statistical significance of specific functional pathways in neuroimaging gene expression associations. Specifically, the enrichment analyses were conducted separately using the significant genes contributing to the first PLS component (PLS1+ and PLS1-) with the following procedures. First, we defined the category score as the mean loading of the genes in the category, and the loading of each gene were calculated based on Spearman correlation between regionalized gene expression profiles and PLS1 score. Secondly, spherical rotation permutations were performed to construct a null model distribution (5000 permutations). Gene loadings were recalculated using the permuted group-difference t-value matrix, generating a null model score distribution at the pathway level. Finally, a two-tailed test was applied to calculate the p-value of the observed pathway score relative to the permutation distribution. The Benjamini-Hochberg false discovery rate correction method was used for multiple comparison correction. The analysis results indicate that this method can effectively identify functional pathways significantly enriched in both the PLS1+ and PLS1-. Pathways with p_fdr < 0.05 demonstrate robust statistical significance (as details in Supplementary Sheet S7).

**Supplementary tables**

**Table S1 Details of Sagittal 3D high-resolution T1-weighted images**

| **Parameters** | **Details** |
| --- | --- |
| Position | sagittal |
| Slices | 188 |
| Slice Thickness | 1.0 |
| Voxel size | 1.0mm×1.0mm×1.0mm |
| Read FOV | 256 |
| Phase FOV | 230 |
| Read Resolution | 256 |
| Phase Resolution | 100 |
| Phase encoding direction | A>P |
| Time Repetition | 6.7 |
| Time Echo | 2.9 |
| Flip angle | 8 |
| Time Inversion | 950 |
| Avarage number | 1 |
| Bandwidth | 270 |
| Echo Train Length | 247 |

**Table S2 Details setting of the BrainSpace toolbox**

| **Parameters** | **Details** | **Explain** |
| --- | --- | --- |
| kernel | cs | cosine similarity |
| approach | dm | default mapping |
| alignment | Procrustes | - |
| n_components | 1 | the number of gradient |
| sparsity | 90 | - |
| alpha | 0.5 | - |
| Diffusion_time | 0 | - |

**Table S3 Details of Neurotransmitters in Juspace**

| **Neurotransmitter maps** | **Name** |
| --- | --- |
| 5HT1a_WAY_HC36.nii | 5HT1a |
| 5HT1a_cumi_hc8_beliveau.nii | 5HT1a* |
| 5HT1b_P943_HC22.nii | 5HT1b |
| 5HT1b_az_hc36_beliveau.nii | 5HT1b* |
| 5HT2a_ALT_HC19.nii | 5HT2a |
| 5HT2a_cimbi_hc29_beliveau.nii | 5HT2a* |
| 5HT4_sb20_hc59_beliveau.nii | 5HT4 |
| 5HT6_gsk215083_HC30_radhakrishnan2018.nii | 5HT6 |
| A4B2_flubatine_HC30_hillmer2016.nii | A4B2 |
| CB1_FMPEPd2_hc22_laurikainen.nii | CB1 |
| CB1_omar_HC77_normandin2015.nii | CB1* |
| CBF_ASL_MRI.nii | CBF |
| CMRglu_fdg_HC20_castrillon2023.nii | CMRglu |
| COX1_ps13_HC11_kim2020.nii | COX1 |
| D1_SCH23390_c11.nii | D1 |
| D2_RACLOPRIDE_c11.nii | D2 |
| D2_fallypride_hc49_jaworska.nii | D2* |
| DAT_DATSPECT.nii | DAT |
| FDOPA_f18.nii | FDOPA |
| GABAa5_ro154513_HC10_lukow2022.nii | GABAa5 |
| GABAa_FLUMAZENIL_c11.nii | GABAa |
| GABAa_flumazenil_hc16_norgaard.nii | GABAa* |
| HDAC_martinostat_HC8_wey2016.nii | HDAC |
| KOR_ly2795050_HC28_vijay2018.nii | KOR |
| KappaOp_LY2795050_hc10_ShokriKojori.nii | KappaOp |
| M1_lsn3172176_HC24_naganawa2020.nii | M1 |
| MOR_carfentanil_HC204_kantonen2020.nii | MOR |
| MOR_carfentanil_HC39_turtonen2021.nii | MOR* |
| MU_CARFENTANIL_c11.nii | MU |
| MU_carfentanil_hc39_turtonen.nii | MU* |
| Microglia_TSPO_pbr28_HC6_lois2018.nii | Microglia |
| PETatlas/NAT_MRB_c11.nii | NAT |
| NMDA_ge179_29hc_galovic2021.nii | NMDA |
| SERT_DASB_HC30.nii | SERT |
| SERT_MADAM_c11.nii | SERT* |
| SERT_dasb_hc100_beliveau.nii | SERT* |
| SV2A_ucbj_HC10_finnema2016.nii | SV2A |
| VAChT_feobv_hc18_aghourian.nii | VAChT |
| VAChT_feobv_hc4_tuominen.nii | VAChT* |
| VAChT_feobv_hc5_bedard.nii | VAChT* |
| VMAT2_dtbz_HC76_larsen2020.nii | VMAT2 |
| mGluR5_abp_hc22_rosaneto.nii | mGluR5 |
| mGluR5_abp_hc28_dubois.nii | mGluR5* |
| mGluR5_abp_hc73_smart.nii | mGluR5* |

*Indicating that there were previous occurrences and repeated neurotransmitters

**Table S4 Demographic and clinical characteristics of the subjects**

| Variables | NSSI (*n* = 134) | Non-NSSI (*n* = 107) | *p* value |
| --- | --- | --- | --- |
| Gender (M/F) | 24/110 | 38/69 | <0.01^a^ * |
| Age (years) | 15.41 ± 1.98 | 16.53 ± 2.21 | <0.01^b^ * |
| TIV (mm^3^) | 1.44×10⁶± 1.85×10⁵ | 1.48×10⁶± 1.82×10⁵ | 0.10^b^ |
| ICD-10 (F32.200/F32.300) | 75/59 | 44/63 | 0.03^a^ * |
| HDRS-17 | 26.57 ± 7.36 | 27.03 ± 5.71 | 0.60^b^ |

^a^ The *p* value is obtained by Chi-square test.

^b^ The *p* value is obtained by two-sample *t*-test.

Data of the continuous variables are shown as mean ± SD.

Statistical differences are marked with *.

F32.2000: Severe depressive episode without psychotic symptoms.

F32.3000: Severe depressive episode with psychotic symptoms.

**Table S5 global residual analysis results**

| **statistic** | **value** |
| --- | --- |
| count | 241 |
| mean | 0.001 |
| std | 0.076 |
| minimum value | -0.129 |
| maximum value | 0.129 |
| median | 0.030 |
| skewness | -0.143 |

Residual distribution: Median-Mean=2.97e-02.

Skewness (-0.14) absolute value <0.5, indicating good symmetry of distribution.

No significant outliers.

**Table S6 GLM model summary**

| coef | std | err | t | P>\|t\| | 95%CI [0.025 0.975] |
| --- | --- | --- | --- | --- | --- |
| Intercept | -0.0781 | 0.097 | -0.810 | 0.419 | -0.268 0.112 |
| sex[T.2] | 0.1029 | 0.103 | 0.998 | 0.319 | -0.100 0.306 |
| age | 0.0043 | 0.006 | 0.762 | 0.447 | -0.007 0.016 |
| age:sex[T.2] | -0.0049 | 0.006 | -0.786 | 0.433 | -0.017 0.007 |
| TIV* | 1.486e-08 | 3.25e-08 | 0.457 | 0.648 | -4.92e-08 7.9e-08 |

*TIV is a covariate used for validation.

**Table S7 Regional MIND gradient differences between NSSI and non-NSSI**

| **Region** | **Name** | **Coordinates** | **t-value** | **p-value** | **Significance** |
| --- | --- | --- | --- | --- | --- |
| 7 | lh_caudalmiddlefrontal_part4 | -39,11,48 | 2.33 | 0.020 | * |
| 25 | lh_inferiortemporal_part2 | -50,-60,-7 | 2.93 | 0.004 | ** |
| 47 | lh_lingual_part3 | -16,-52,-6 | -2.92 | 0.004 | ** |
| 55 | lh_middletemporal_part2 | -56,-58,2 | 1.98 | 0.049 | * |
| 180 | rh_inferiortemporal_part2 | 51,-58,-8 | 2.33 | 0.021 | * |
| 279 | rh_superiorparietal_part1 | 23,-81,24 | -2.70 | 0.007 | ** |

Note: A GLM was used to investigate regional MIND gradient alterations in NSSI group, while regressing out the effect of age, sex and age × sex. The *t*-statistic > 0 means non-NSSI > NSSI, and the *t*-statistic < 0 means non-NSSI < NSSI. All *p*-values survived after Cluster-FDR correction with *p* < 0.05. Here, * indicates p < 0.05, and ** indicates p < 0.01.

**Table S8 The results of the Yeo-7 network**

| **Network** | **t-value** | **p-value** |
| --- | --- | --- |
| VN | -1.054 | 0.293 |
| SMN | 0.050 | 0.956 |
| DAN | 1.781 | 0.076 |
| VAN | 0.782 | 0.439 |
| LN | 0.882 | 0.382 |
| FPN | 0.970 | 0.334 |
| DMN | 1.071 | 0.287 |

**Table S9 The results of the von Economo network**

| **Network** | **t-value** | **p-value** |
| --- | --- | --- |
| Prim motor | -0.552 | 0.582 |
| Asso1 | 1.284 | 0.201 |
| Asso2 | 0.750 | 0.453 |
| Sec sens | -0.992 | 0.322 |
| Prim sens | -1.012 | 0.314 |
| Limbic | 0.700 | 0.485 |
| Insula | 0.700 | 0.488 |

**Table S10 Cluster statistics under different thresholds**

| threshold | n_clusters | regions | avg_cluster_size | median_cluster_size | coverage_percentage |
| --- | --- | --- | --- | --- | --- |
| 5 | 5 | 6 | 1.2 | 1 | 1.948 |
| 10 | 5 | 6 | 1.2 | 1 | 1.948 |
| 15 | 5 | 6 | 1.2 | 1 | 1.948 |
| 20 | 5 | 6 | 1.2 | 1 | 1.948 |

**Table S11 Regional MIND gradient differences of Group1 and Group2**

| **Group1** | | | | | |
| --- | --- | --- | --- | --- | --- |
| **Region** | **Name** | **Coordinates** | **t-value** | **p_fdr** | **Significance** |
| 25 | lh_inferiortemporal_part2 | -50,-60,-7 | 2.23 | 0.027 | * |
| 47 | lh_lingual_part3 | -16,-52,-6 | -2.47 | 0.027 | * |
| 180 | rh_inferiortemporal_part2 | 51,-58,-8 | 2.34 | 0.027 | * |
| 279 | rh_superiorparietal_part1 | 23,-81,24 | -2.86 | 0.019 | * |
| **Group2** | | | | | |
| **Region** | **Name** | **Coordinates** | **t-value** | **p_fdr** | **Significance** |
| 7 | lh_caudalmiddlefrontal_part4 | -39,11,48 | 2.28 | 0.035 | * |
| 25 | lh_inferiortemporal_part2 | -50,-60,-7 | 2.59 | 0.032 | * |
| 47 | lh_lingual_part3 | -16,-52,-6 | -2.51 | 0.032 | * |
| 198 | rh_lateralorbitofrontal_part4 | 19,30,-19 | 2.12 | 0.035 | * |
| 279 | rh_superiorparietal_part1 | 23,-81,24 | -2.13 | 0.035 | * |

Note: A GLM was used to investigate regional MIND gradient alterations in NSSI group, while regressing out the effect of age, sex and age × sex. The *t*-statistic > 0 means non-NSSI > NSSI, and the *t*-statistic < 0 means non-NSSI < NSSI. All *p*-values survived after Cluster-FDR correction with *p* < 0.05. Here, * indicates p < 0.05.

**Supplementary figures**

**Fig. S1 Elaboration of MIND.** Firstly, the surface was subdivided into 308 contiguous areas based on the D-K 308 atlas. Each area was characterized by five structural features: surface area (SA), cortical thickness (CT), mean curvature (MC), gray matter volume (GMV) and sulcal depth (SD). To estimate the similarity between cortical regions, we normalized each MRI feature across all vertices and then summarized all MRI measures across all vertices within each cortical region. Furthermore, the K-nearest neighbor density algorithm was used to compile a paired distance matrix to estimate the KL divergence between each pair of regional multivariate distributions. Finally, the KL divergence KL (a, b) of regions a and b was transformed to estimate the MIND similarity between regions. The MIND was bounded between 0 and 1, with higher values indicating greater similarity.

**Fig. S2 Spatial cluster connectivity rule.**

**Fig. S3. Euler number box plot between two groups.** Left hemisphere: Group1: mean: -46.953, standard deviation: 20.356, median: -44.000. Group2: mean: -46.881, standard deviation: 16.660, median: -44.000. The independent sample t-test results showed a t-value of -0.030 and a p-value of 0.976, with no significant difference between the two groups (p>=0.05). Right hemisphere: Group1: mean: -41.308, standard deviation: 17.836, median: -38.000. Group2: mean: -40.985, standard deviation: 14.990, median: -40.000. The independent sample t-test results showed a t-value of -0.150 and a p-value of 0.881, with no significant difference between the two groups (p>=0.05). Whole brain: Group1: mean: -88.262, standard deviation: 35.068, median: -82.000. Group2: mean: -87.866, standard deviation: 28.603, median: -82.000. The independent sample t-test results showed a t-value of -0.094 and a p-value of 0.925, with no significant difference between the two groups (p>=0.05). Group1: without NSSI, Group2: NSSI.

**Fig. S4. Box plot of group comparision across Yeo-7 network and von Economo network.** The above figure shows inter group comparison on the Yeo-7 network: blue (without NSSI) and red (NSSI) bins have different distributions in different brain networks (such as VN, SMN and DAN). Although the bins in the without NSSI group are significantly higher than those in the NSSI group, the median is similar in the VN group. Statistical significance: p=0.0756 in DAN, indicating that the difference between groups is close to statistical significance (marginal significance). No significant outliers were observed, and the data distribution is relatively stable. On the von Economo network below, we did not observe statistically significant differences. And no obvious outliers were observed, indicating a relatively stable data distribution.

**Fig. S5. Relative contribution percentage of PLS1 and PLS2.** We used a mixed strategy of 10000 bootstrap samples combined with multiple linear regression to systematically evaluate the significant contribution of PLS1 and PLS2 scores to the group-difference t-value. First, generate 10000 subsets of data through resampling with replacement; Subsequently, a multiple linear regression model was established with group-difference t-value as the dependent variable and PLS1 and PLS2 scores as independent variables to obtain their independent contribution coefficients to the t-value; Finally, based on the coefficient distribution of 10000 regression results, calculate the confidence interval and p-value, and determine the contribution proportion according to the percentage of the absolute value of the standardized coefficient to the total absolute value.

**Fig. S6. Validation of TIV effect on group-difference principal MIND gradient differences. a.** The group-difference *t* map with controlling for TIV. **b.** The group-difference *t* map without controlling for TIV. **c.** The results of spatial correlation analysis between the principal MS gradient with and without controlling for TIV. *P* value was calculated based on spin test.

**Fig. S7.** **The effect of different types of correlation analysis on group differences.** **A.** The group-difference *t* map of the principal MIND gradient based on Spearman’s correlation analysis. **B.** The group-difference *t* map of the principal MIND gradient based on Pearson’s correlation analysis. **C.** The results of spatial correlation analysis between the principal MIND gradient based on Spearman’s and Pearson’s correlation analysis.

**Fig. S8.** **Evaluating the network topology characteristics under different cluster thresholds** **size.** It was found that the statistical results of group-difference MIND Gd differences remained highly consistent at each threshold (n=5, 10, 15, 20). The clustering distribution patterns, node connection characteristics, and global topology indicators obtained from different threshold settings showed no significant statistical differences.

**Fig. S9. Comparison of principal MIND gradient differences between NSSI subgroups and without NSSI. (a, c)** Group1 revealed principal MIND gradient differents in the left inferior temporal (part2), left lingual (part3), right inferior temporal (part2) and right superior parietal (part1), while Group2 in the left caudal middle frontal (part4), left inferior temporal (part2), left lingual (part3), right lateral orbitofrontal (part4) and right superior parietal (part1), as detailed in Supplementary Table S11. Except for right lateral orbitofrontal region, the remaining brain regions in both groups overlap with those in the entire NSSI group.The overlap rates of differential regions in both two subgroups reach up to 3/5. **(b, d)** The group-difference t-map for NSSI patients displayed positive spatial correlations with that of Group1 (r =0.912, *p*_spin_ =0.002, spin=10000) and Group2 (r =0.933, *p*_spin_ <0.001, spin=10000).

**Fig. S10. Sex-stratified analysis of NSSI-related MIND-Gd group differences.** Node-wise t-values from GLM (group difference adjusted for age and TIV) in **(a)** females (n=179; NSSI 110 vs non-NSSI 69) and **(b)** males (n=62; NSSI 24 vs non-NSSI 38) plotted against the full-sample t-map across 308 DK-308 regions. Female (r=0.793, p_spin_=0.0032) and male (r=0.374, p_spin_=0.0078) t-maps were each significantly correlated with the full-sample pattern (spin test with 10,000 permutations; dashed line, y=x). The smaller male subgroup warrants caution in interpreting the male pattern.

**Fig. S11. Leave-one-donor-out (LODO) sensitivity analysis for the PLS1-based transcriptomic association.** Bars show the spatial Spearman correlation between PLS1 scores and the group-difference t-map, recomputed after excluding each of the six Allen Human Brain Atlas (AHBA) donors individually. The dashed red line indicates the full-model correlation (r = 0.557). All LODO models remained significant under 10,000 spatial permutations (all p_spin_ < 0.001), with correlations ranging from r = 0.539 to 0.563, indicating that no single donor disproportionately drove the observed PLS1 spatial pattern.
